# The first chromosome-level genome of the stag beetle *Dorcus hopei* Saunders, 1854 (Coleoptera: Lucanidae)

**DOI:** 10.1038/s41597-024-03251-x

**Published:** 2024-04-18

**Authors:** Xiaolu Li, Chuyang Mao, Jinwu He, Xiaoyan Bin, Guichun Liu, Zhiwei Dong, Ruoping Zhao, Xia Wan, Xueyan Li

**Affiliations:** 1 Department of Ecology, School of Resources and Environmental Engineering, Anhui University, Hefei, 230601 China; 2 Key Laboratory of Genetic Evolution & Animal Models, Kunming Institute of Zoology, Chinese Academy of Sciences (CAS), Kunming, Yunnan 650223 China; 3 Yunnan Key Laboratory of Biodiversity Information, Kunming, Yunnan 650223 China

**Keywords:** Genome, Entomology

## Abstract

Stag beetles (Coleoptera: Lucanidae) represent a significant saproxylic assemblage in forest ecosystems and are noted for their enlarged mandibles and male polymorphism. Despite their relevance as ideal models for the study of exaggerated mandibles that aid in attracting mates, the regulatory mechanisms associated with these traits remain understudied, and restricted by the lack of high-quality reference genomes for stag beetles. To address this limitation, we successfully assembled the first chromosome-level genome of a representative species *Dorcus hopei*. The genome was 496.58 Mb in length, with a scaffold N50 size of 54.61 Mb, BUSCO values of 99.8%, and 96.8% of scaffolds anchored to nine pairs of chromosomes. We identified 285.27 Mb (57.45%) of repeat sequences and annotated 11,231 protein-coding genes. This genome will be a valuable resource for further understanding the evolution and ecology of stag beetles, and provides a basis for studying the mechanisms of exaggerated mandibles through comparative analysis.

## Background & Summary

Stag beetles (Family: Lucanidae), comprise over 1,800 species and subspecies^1^, noted for enlarged allometry mandibles and male polymorphism^2^. As a holometabolous insect, they undergo a complete metamorphosis with four life stages: egg, larva, pupa, and adult^3,4^. The larvae primarily feed on decaying wood, while the adults are mostly nocturnal and feed on plant juices, fruits, or other decaying organic matter^5–7^. Due to their saprophagous nature, stag beetles play essential roles in the carbon and nitrogen cycles, which also makes stag beetles an important indicator species for evaluating forest ecosystems^1,8,9^. Based on observations of male lucanid beetles, Darwin (1871)^10^ noted that “The great mandibles of the male Lucanidae are extremely variable both in size and structure…and are used as efficient weapons for fighting”. It implies that individuals with larger mandibles have a better chance of defeating their rivals and winning mating rights^11,12^. Due to its unique and variable appearances, as well as interesting behavioral phenomena, this group has garnered the affection of many collectors, entomologists and evolutionary biologists^8,10^.

Stag beetles are widely distributed across various biogeographic regions and represent a group with significant nodal importance in the process of evolution^13^. The research on the Lucanidae family is predominantly concentrated on molecular taxonomic studies^8,14,15^, based on the data from nuclear gene fragments^16^, mitochondrial multi-gene fragments^17^ and mitochondrial genomes^18,19^. However, these data are insufficient to provide more insights into the formation and differentiation of the stag beetles’ mandibles^20^. Decoding high-quality reference genomes has been proven to be the cornerstone of inferring phylogeny and exploring the molecular basis behind phenotypic innovation^21^, e.g., the antlers of cervids^22^, the long tail feathers of birds^23,24^, and the horns of some scarabs^25,26^. The limited availability of genomic data hindered our research on Lucanidae family.

**Fig. 1:**
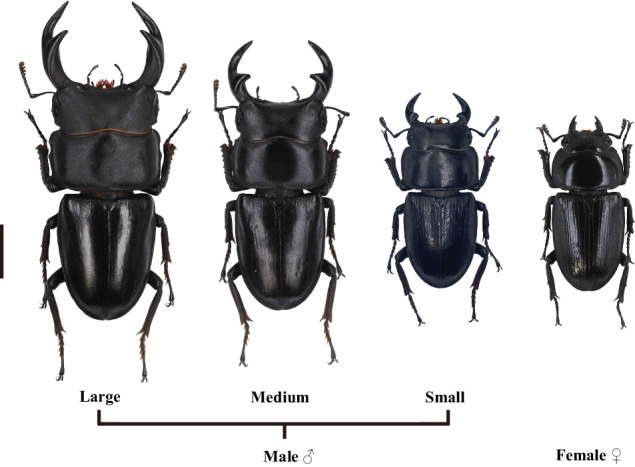
Sexual dimorphism and male trimorphism in *Dorcus hopei*. The scale bar is 1 cm.

*Dorcus hopei* (Saunders, 1854), distributing from central and northeastern China, is a well-known species notable for its sword-shaped mandibles^2,27,28^ (Fig. 1). Comparatively, its mandibles are simpler to observe, with a large sharp bump and a relatively small inner tooth. Owing to the restriction of insect allometry and scaling relationship^29–31^, its male trimorphism in mandibles and body sizes is a very rare type. Based on these characteristics, *D. hopei* is a good choice for performing long-term studies in stag beetles.

In this study, we successfully assembled the first chromosome-level reference genome of *D. hopei* using Illumina, Nanopore and Hi-C sequencing, the information from which could enhance our understanding of stag beetle survival and evolution. Furthermore, it provides a novel clue for uncovering the molecular basis of extreme mandibles development and male trimorphism formation in the future.

## Methods

### Sample information

Adult male *D. hopei* specimens were collected from Shou County, Huainan City, Anhui Province, China, during May and June from 2017 to 2021. The beetles were subsequently reared in the laboratory (23 °C, 14 h:10 h light/dark cycle, and 45% relative humidity) and provided with brown sugar jelly and bananas as food. Two adult males were selected for next-generation genomic sequencing using the Illumina platform, one adult male was used for long-read genomic sequencing with the Oxford Nanopore platform, two adult males were selected for Hi-C sequencing, and one adult male was used for transcriptomic sequencing.

### Illumina, nanopore, Hi-C, and RNA sequencing

Genomic DNA was isolated from the leg muscles using a Trelief Animal Genomic DNA Kit (TsingKe, China). Paired-end libraries (insert size: 350 bp) were generated using a NEBNext Ultra DNA Library Prep Kit (New England Biolabs, USA) with the Illumina HiSeq 4000 platform at Novogene (Tianjin, China). After filtering the bases in the raw reads of quality <Q20, we obtained 55.13 Gb (113x) clean Illumina data.

For Oxford Nanopore long-read sequencing, DNA from thorax muscles were extracted using a Qiagen DNAeasy Kit (Qiagen, German). Subsequently, the extracted DNA was treated with the NEBNext Ultra End Repair/dA-Tailing module (New England Biolabs, USA) to incorporate adapters for priming sequencing reactions (NextOmics, China). The library was constructed using a 1D DNA Ligation Sequencing Kit (SQK-LSK109) (Oxford Nanopore Technologies, England) and sequencing was performed on a PromethION flow cell (NextOmics, China) to obtain 46.34 Gb (95x) Nanopore data.

For Hi-C sequencing, cells isolated from head tissues were fixed with formaldehyde and subsequently digested using the restriction enzyme MboI. The DNA was purified and then sheared into 300–600 bp fragments using a Covaris M220 device (Covaris, USA). After DNA size selection using AMPure XP beads, point ligation junctions were pulled down using Dynabeads MyOne Streptavidin C1 (ThermoFisher, USA). Then the Hi-C library was sequenced on the Illumina NovaSeq sequencing platform at Novogene (China), and we got 48.46 Gb (100x) Hi-C data.

Transcriptomic sequencing was used to assist in gene structure annotation. RNA was extracted from the head tissue of one adult male using TRIzol. RNA quality was assessed using an RNA Nano 6000 Assay Kit for 2100 Bioanalyzer Systems Kit (Agilent Technologies, China). The libraries were generated using a NEBNext Ultra RNA Library Prep Kit (New England Biolabs, USA) and sequenced on the Illumina Hiseq platform at Novogene (Tianjin, China). And 8.07 Gb were obtained for assisting genomic annotation.

### Chromosome-level genome assembly

**Fig. 2:**
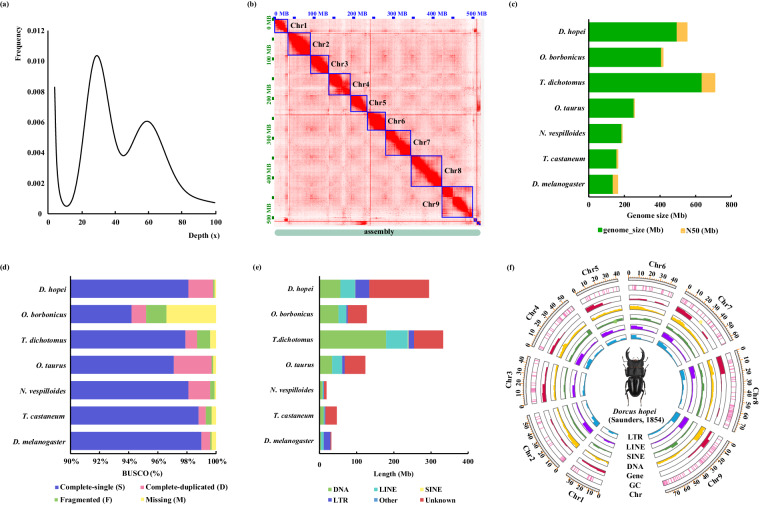
Assembly of chromosome-level genome of *Dorcus hopei*. (**a**) 17-mer analysis of the *D. hopei* genome based on Illumina reads, X-axis represented depth (x); Y-axis represented the proportion of the frequency of that depth to the total frequency of all depths. (**b**) Heatmap of Hi-C data showing nine chromosome boundaries (Chr1 to Chr9). The comparison of (**c**) genome size and N50 length, (**d**) BUSCO scores, and (**e**) repeat elements in *D. hopei* and six other species. (**f**) Circos tracks showing chromosome length, GC content, density of protein-coding genes, and repetitive elements (SINE, short interspersed elements; LINE, long interspersed elements; LTR, long terminal repeat elements).

Illumina data was used to estimate genome size based on 17 *k-mer* size analysis using KmerFreq v5.0^32^. The estimated genome size of *D. hopei* was 487.15 Mb, with heterozygosity of 0.021 based on the frequency distribution of 17-mers (Fig. 2a). The Oxford Nanopore long reads were used to assemble and polish the primary genome with NextDenovo v2.5.0 (https://github.com/Nextomics/NextDenovo) (parameters: -k 0 -p 15) and purge_dups v1.0.0^33^. The Illumina short reads were used to correct errors at the base level in the above-polished genome using NextPolish v1.4.0^34^. The Nanopore assembly was 496.47 Mb (N50 = 3.94 Mb), comprised 232 contigs, and achieved a BUSCO completeness score of 99.90% (Table 1).

**Table 1:** Statistics for *Dorcus hopei* genome assembly and gene annotation.

Statistics	Nanopore assembly	Hi-C assembly
Assembly		
Total number	232	27
Genome size (Mb)	496.47	496.58
Average length (Mb)	2.14	18.39
N50 length (Mb)	3.94	54.61
N90 length (Mb)	1.01	33.12
Maximum length (Mb)	17.85	75.00
GC content (%)	35.23	35.22
BUSCO (completeness, %)	99.9	99.8
Anchor rate (%)	—	96.18
Illumina reads mapping rate (properly paired) (%)	99.10 (94.20)	99.10 (94.20)
Nanopore reads mapping rate (%)	88.81	100.00
Annotation		
Gene number		11,231
Average gene length (bp)		15,840.00
Average CDS^a^ length (bp)		1,714.51
Average exon number		6.58
Average exon length (bp)		260.59
Average intron length (bp)		2,531.69
BUSCO (%)		92.0

^a^CDS: coding sequence.

The Hi-C paired-end reads were iteratively mapped to the Nanopore assembly using HiC-Pro v2.9.0^35^. The paired tags were then filtered using restriction enzyme digesting fragments with Juicer v1.60 and contigs were ordered and orientated using 3D *de novo* assembly software (3D-DNA) v180922^36^. Finally, JuiceBox v1.11.08^37^ was applied to correct contig orientation and move suspicious fragments into unanchored groups by visual exploration of the Hi-C heatmap. After Hi-C assembly, the resulting 496.58 Mb genome was assembled into 18 chromosomes (2n = 8AA + XY) (Table 1; Fig. 2b). Notably, 96.18% of the contigs from the “Nanopore assembly” were successfully anchored to nine chromosomes, with a scaffold N50 of 54.61 Mb and 99.80% BUSCO completeness (1.7% duplicated genes) (Table 1), indicating relatively high assembly integrity (Fig. 2c,d).

### Genome annotation

We choose several reference species to assist annotation, including five other coleopteran species (Scarabaeoidea: *Onthophagus taurus* (GCA_000648695.2), *Oryctes borbonicus*^38^ (GCA_902654985.2), *Trypoxylus dichotomus*^39^ (GCA_023509865.1); Staphylinidea: *Nicrophorus vespilloides*^40^ (GCA_001412225.1); Tenebrionoidea: *Tribolium castaneum*^41^ (GCA_000002335.3)), and one dipteran species, *Drosophila melanogaster*^42^ (GCA_000001215.4). We uploaded the detailed species information table to figshare^43^. Initially, we annotated repetitive sequences in the *D. hopei* genome by identifying LTRs and tandem repeats using LTR_Finder v1.05^44^ and Tandem Repeat Finder v4.07b^45^, respectively. Transposable elements (TEs), including DNA elements, long interspersed nuclear elements (LINEs), short interspersed nuclear elements (SINEs), and long terminal repeats (LTRs), were next identified using RepeatMasker v4.0.5^46^ against a *de novo* repeat library constructed with RepeatModeler v1.0.4^47^ and Repbase TE library v16.02^48^ separately at the DNA level. Finally, TE-relevant proteins were identified using RepeatProteinMask v4.0.9^47^ at the protein level. The final genome assembly (Hi-C assembly) of *D. hopei* comprised 57.45% repetitive sequences, totaling approximately 285.27 Mb, which is almost twice that of *T. castaneum* (31.15%) (Fig. 2e). Among the repetitive sequences in the *D. hopei* genome, the major categories included unclassified sequences (32.39%), DNA elements (11.36%) with maximum density in each chromosome, LINEs (7.95%), and LTRs (7.55%) (Fig. 2f).

**Fig. 3:**
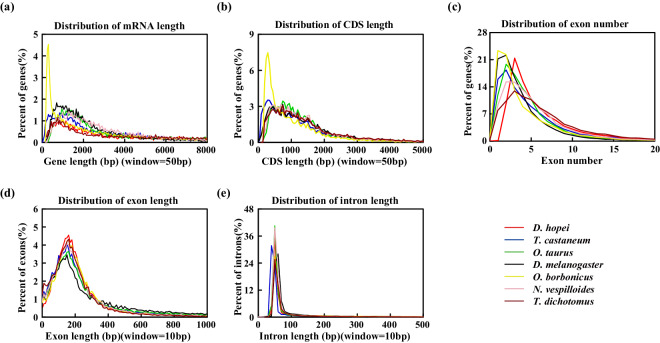
Distribution statistics of gene features among the seven species. The comparison of (**a**) mRNA length, (**b**) CDS length, (**c**) exon number, (**d**) exon length and (**e**) intron length in *D. hopei* and other six species.

Protein-coding genes were predicted using a combination of *de novo*-, homology-, and transcriptome-based approaches. We utilized the repeat-masked genome and applied the *de novo*-based gene prediction software Augustus v3.4.0^49^, using models trained on protein sequences from the *O. borbonicus* genome^38^, with default parameters. TBLASTN v2.12.0^50^ and GeneWise v2.4.1^51^ were used for homology prediction. The transcriptome data were then aligned to the genome using HISAT2 v2.0.0-beta^52^. Based on the resulting BAM files and reference genome, the transcriptomic sequences were assembled using StringTie v2.1.4^53^. To form a comprehensive, non-redundant set of genes, we performed several integrations using EVidenceModeler (EVM) v1.1.1^54^, assigning different weight values to the seven genomes based on their BUSCO scores and gene structure components (gene length, coding sequence length, exon number and length, and intron length). The EVM gene set with the best BUSCO value and gene structure components was then selected as the final gene prediction. Finally, resulting in the annotation of 11,231 protein-coding genes in the *D. hopei* genome. We uploaded the complete gene annotation tables to figshare^43^. Compared to the different gene features of other six species, the *D. hopei* genome annotations were comprehensive (Fig. 3), further validating the quality and accuracy of the genome annotation.

Finally, we performed functional annotation of the genome. The protein sequences of the genome were searched for homology-based function assignments against the KEGG, NR, TrEMBL, and SwissProt databases using BLASTP v2.2.26^55^ with an e-value cut-off of 1e-5. Domains in the *D. hopei* genome using InterProScan v5.54–87.0^56^ with InterPro and GO database. And combined above results, 88.52% of the predicted genes were functionally annotated using six functional protein databases (Table 2).

**Table 2:** Statistics of functional annotation of the *Dorcus hopei* protein-coding genes.

Functional database	Number of genes annotated (11,231)
Annotated	9,942 (88.52%)
Unannotated	1,289 (11.48%)
InterPro	8,737 (77.79%)
GO	8,737 (77.79%)
KEGG	6,556 (58.37%)
SwissProt	7,654 (68.15%)
TrEMBL	9,779 (87.07%)
NR	9,838 (87.60%)

## Data Records

The chromosome-level assembly and annotation file of *D. hopei* has been deposited in figshare database^57^. Raw sequencing data (Illumina reads, Nanopore reads, Hi-C reads, RNA-seq reads) and sample information are available at NCBI, which can be found under identification number SRP440764^58^. The assembly also has been deposited in NCBI with the accession number GCA_033060865.1^59^. More detailed information about selected species, the results of genomic annotation (repeated sequences and gene structure), orthologs, and synteny has been deposited in figshare database^43^.

## Technical Validation

Quality assessment of the assembled genome was performed using the following methods. Firstly, BWA v0.7.17^60^ was used to map the Illumina reads to the *D. hopei* assembly and Samtools v1.3.1^61^ was used to calculate the mapping ratio. The Illumina short reads with a 99.10% accuracy ratio were mapped to the final assembly (Table 1). Secondly, compared N50 length/number with other six selected species. The *D. hopei* genome displayed a longer N50 (54.61 Mb) and better continuity compared to the chromosome-level genomes of *T. castaneum* and *T. dichotomus* (Fig. 2c). Thirdly, insecta_odb10 with 1,367 genes in BUSCO v5.2.2^62^ was used to evaluate genome assembly and annotation completeness. The final assembly had 99.8% BUSCO scores with 0.1% fragmented and 0.1% missing sequences (Fig. 2d). Additionally, we got nine pairs of chromosomes based on Hi-C data, mirroring that of congeneric species *Dorcus parallelipipedus*^63^. All these results suggest that we got a high-quality assembly of *D. hopei* with high integrity, continuity and accuracy.

## Data Availability

No custom code was used in this study. The data analyses used standard bioinformatic tools specified in the methods.
